# Supplementary material for: Single-photon decision maker
Source: Sci Rep. 2015 Aug 17;5:13253. doi: 10.1038/srep13253 (PMC4538607; doi:10.1038/srep13253)
Supplement: Supplementary Information [file srep13253-s1.pdf]

Supplementary Information

Single-photon decision maker

Makoto Naruse[1], Martin Berthel[2,3], Aurélien Drezet[2,3], Serge Huant[2,3], Masashi Aono[4,5], Hirokazu Hori[6] & Song-Ju Kim[7]

1 Photonic Network Research Institute, National Institute of Information and Communications Technology, 4-2-1 Nukui-kita, Koganei, Tokyo 184-8795, Japan

2 Université Grenoble Alpes, Inst. NEEL, F-38000 Grenoble, France

3 CNRS, Inst. NEEL, F-38042 Grenoble, France

4 Earth-Life Science Institute, Tokyo Institute of Technology, 2-12-1 Ookayama, Meguru-ku, Tokyo 152-8550, Japan

5 PRESTO, Japan Science and Technology Agency, 4-1-8 Honcho, Kawaguchi-shi, Saitama 332-0012, Japan

6 Interdisciplinary Graduate School of Medicine and Engineering, University of Yamanashi, Takeda, Kofu, Yamanashi 400-8511, Japan

7 WPI Center for Materials Nanoarchitectonics, National Institute for Materials Science, 1-1 Namiki, Tsukuba, Ibaraki 305-0044, Japan

Second-order photon-intensity correlation measurements

The image shown in Supplementary Fig. 1a is a confocal microscope image of the single NV centre used in the main text, and the light blue curve in Supplementary Fig. 1b shows the result of measuring the second-order photon correlation from the single NV centre. The measurement was made using a standard Hanbury–Brown and Twiss correlator. The thick red curve is a fit with a three-level model[1]. The antibunching dip, which is a signature of the quantum nature of the emission, does not drop to zero at a finite delay as expected from a single-photon source because the detectors receive some spurious background fluorescence B ≈ 2000 photons/s in addition to the useful (quantum) emission S ≈ 5000 photons/s from the single NV centre. Taking into account this background in the three-level fit as explained in ref. 1 gives an antibunching dip at zero delay dropping to approximately 0.5, in agreement with the experiment.

Dependence of single-photon decision maker on strategy

As discussed in the Methods section, the orientation of the linear polarizer based on the truncated integer values of the PA values are given by, for the **Control 1** policy, $\mathrm{Pos}(-3) = 60$, $\mathrm{Pos}(-2) = 59$, $\mathrm{Pos}(-1) = 58$, $\mathrm{Pos}(0) = 34$, $\mathrm{Pos}(1) = 21$, $\mathrm{Pos}(2) = 20$, and $\mathrm{Pos}(3) = 19$, which we refer to hereafter as **Strategy 1**. Let us define the effective extinction ratio (EER) by the number of the photon counts detected in Ch.0 divided by those detected in Ch.1, which are experimentally measured, as shown in Fig. 4. The correspondence between the truncated PA values and the EER of **Strategy 1** is indicated by the red square marks in the inset of Supplementary Fig. 2, where EER is more sensitive around the origin of the truncated PA value [$\lceil PA(t) \rceil = 0$]. We can think of other two contrasting strategies where (1) EER changes gradually as a function of the truncated PA values (**Strategy 2**) and (2) the change in EER is sensitive at

the boundary of the range of the truncated PA values (**Strategy 3**), which are shown by green circles and blue triangles, respectively. Specifically, **Strategies 2** and **3** are, respectively, defined by

Strategy 2: $\mathrm{Pos}(-3)=60$, $\mathrm{Pos}(-2)=51$, $\mathrm{Pos}(-1)=42$, $\mathrm{Pos}(0)=34$, $\mathrm{Pos}(1)=29$, $\mathrm{Pos}(2)=24$, and $\mathrm{Pos}(3)=19$

Strategy 3: $\mathrm{Pos}(-3)=60$, $\mathrm{Pos}(-2)=36$, $\mathrm{Pos}(-1)=35$, $\mathrm{Pos}(0)=34$, $\mathrm{Pos}(1)=33$, $\mathrm{Pos}(2)=32$, and $\mathrm{Pos}(3)=19$.

The resulting decision-making performance of **Strategies 1, 2,** and **3** in terms of correct selection rate is shown by the red solid, green dashed, and blue dotted curves, respectively, in Supplementary Fig. 2, where no evident difference in performance is observed. Based on this evaluation and by considering a strategy in which EER exhibits a dramatic change around *PA* = 0, we choose **Strategy 1** for the experimental demonstration discussed in the main text.

Supplementary references

1. Berthel, M. *et al.* Photophysics of single nitrogen-vacancy centers in diamond nanocrystals. *Phys. Rev. B* **91**, 035308 (2015).

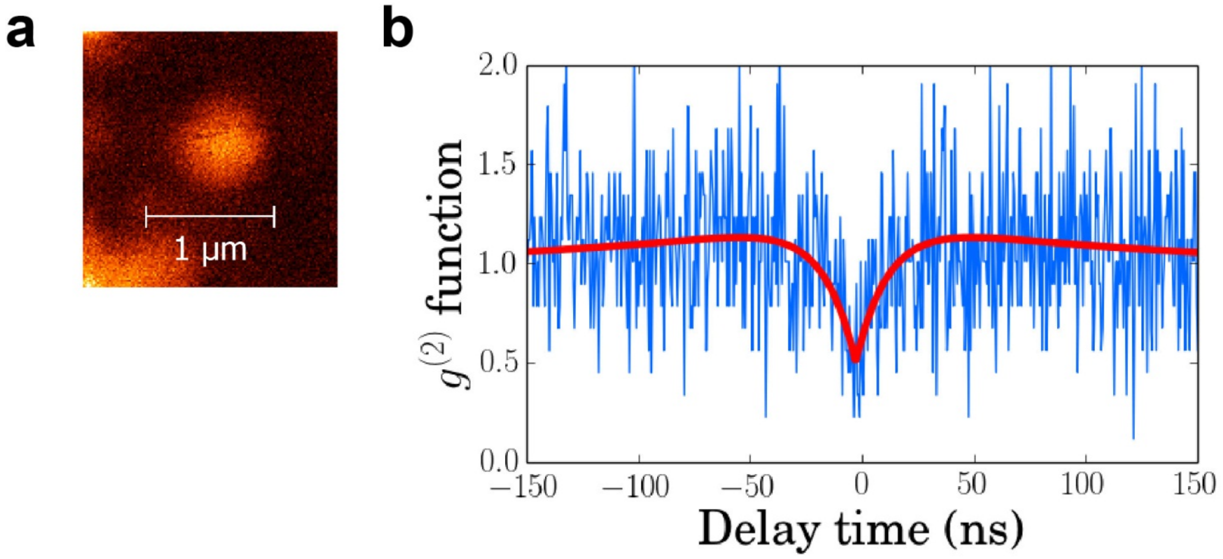

Supplementary Figure 1 | Second-order photon–intensity correlation measurement of the single NV centre. (a) A confocal microscope image of the single NV centre used in the main text. (b) The red curve is fit to three-level model.

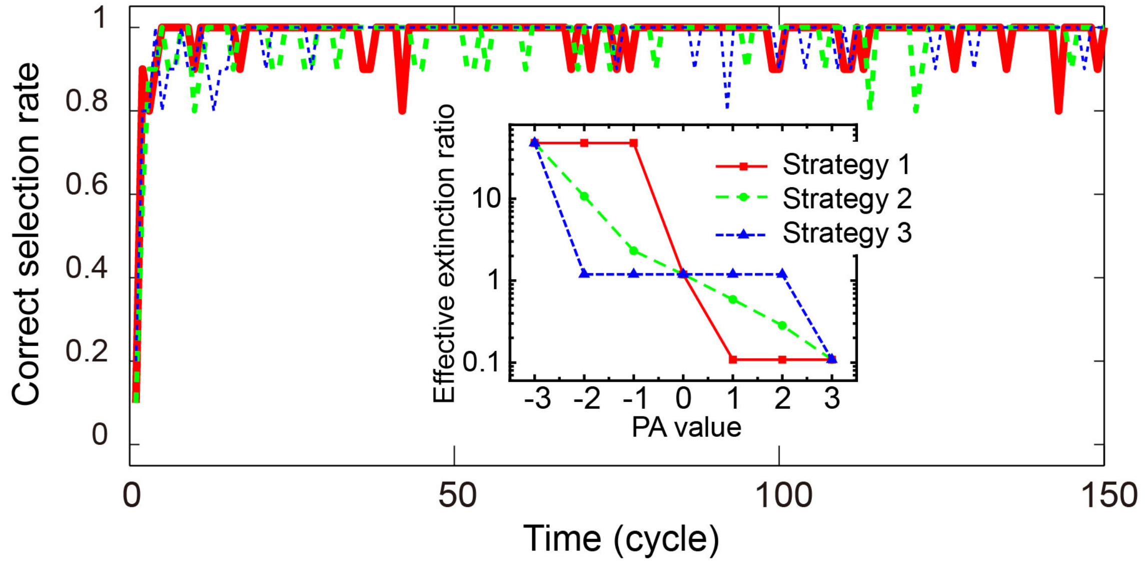

Supplementary Figure 2 | Dependence of single-photon decision maker on strategy. The resulting decision-making performance of **Strategies 1, 2**, and **3** in terms of correct selection rate is shown by the red solid, green dashed, and blue dotted curves.
